# Exploring Oral Polymorphous Adenocarcinoma: Clinical Characteristics, Diagnosis, and Treatment Outcomes. A Case Report

**DOI:** 10.3390/reports8020070

**Published:** 2025-05-16

**Authors:** Christina Charisi, Vasileios Zisis, Petros Papadopoulos, Konstantinos Poulopoulos, Nikolaos Kyriakou, Athanasios Poulopoulos

**Affiliations:** 1 Department of Oral Medicine/Pathology 1, School of Dentistry, Aristotle University of Thessaloniki, 54124 Thessaloniki, Greece; charisidentist@gmail.com (C.C.);

**Keywords:** polymorphous adenocarcinoma, oral, intraoral, palate, salivary gland, salivary malignancies, salivary tumors, oral cancer, head and neck cancer

## Abstract

**Background and clinical significance**: Polymorphous adenocarcinoma of the oral cavity is predominantly located in the palate. It is characterized by a slow rate of growth and thus may be misdiagnosed as a benign tumor. Its histology is intricate with other salivary malignancies, thus necessitating specific immunohistochemical stains. Our case report illustrates an adenocarcinoma localized in the palate of a 61-year-old female patient. **Case presentation:** The patient came to the postgraduate clinic of Oral Medicine and Pathology, Department of Oral Medicine and Pathology, School of Dentistry, Faculty of Health Sciences, Aristotle University of Thessaloniki, Greece and provided written informed consent for the subsequent examination. The patient complained about the presence of a mass on the palate, which was otherwise asymptomatic, without being able to pinpoint when the tumor initially emerged. The lesion was biopsied and the histology suggested the immunophenotype p63+/p40- which constitutes an important diagnostic clue for polymorphous adenocarcinoma. The patient was referred to the Department of Oral and Maxillofacial Surgery. Conclusions: The standard therapeutic approach primarily involves surgical excision. The goal is to achieve optimal patient outcome while minimizing unnecessary morbidity. As surgical techniques and understanding of the disease continue to advance, it is crucial for healthcare providers to stay informed and integrate these developments into practice to improve treatment outcomes for patients.

## 1. Introduction and Clinical Significance

Polymorphous adenocarcinoma of the oral cavity is predominantly located in the palate, highlighting a significant trend, as seen in 10 out of 14 cases [1]. This type of neoplasm is characteristically slow growing, often present for many years before a definitive diagnosis is made [1]. Colmenero et al. identified nine cases, initially misclassified as other benign or malignant tumors, prior to their identification as polymorphous adenocarcinoma [1]. The same authors found only one case located at the base of the tongue, buccal mucosa, tonsil, and retromolar pad [1]. Despite the potential for recurrence, which is noted at a rate of 17%, and the possibility of metastases, particularly with the terminal duct adenocarcinoma type, the prognosis remains relatively favorable, with no deaths related to the neoplasm in the aforementioned cases [1]. As oral polymorphous adenocarcinoma progresses, it manifests invasive characteristics that contribute to its local destructiveness. This leads to perineural invasion, which is observed in approximately 30% of cases [2]. The peripheral expansion leads to infiltration into non-neoplastic seromucous glands, adjacent soft tissues, and bone [2]. Such a growth pattern is typical of malignancy in minor salivary gland neoplasms [2]. As the tumor advances, the surrounding stroma may undergo hyalinization and myxoid change, although it does not develop the chondromyxoid matrix seen in pleomorphic adenoma [2]. Our case report illustrates a p63+/p40- polymorphous adenocarcinoma localized in the palate of a 61-year-old female patient, enriching the related literature.

## 2. Case Presentation

**Figure 1:**
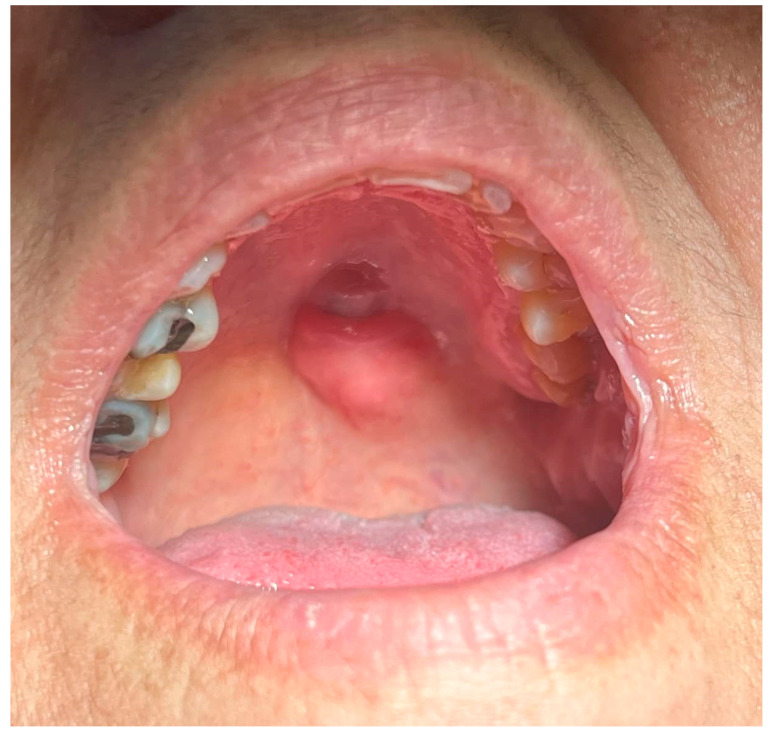
Initial clinical appearance of the tumor.

The 61-year-old female patient came to the postgraduate clinic of Oral Medicine and Pathology, Department of Oral Medicine and Pathology, School of Dentistry, Faculty of Health Sciences, Aristotle University of Thessaloniki, Greece and provided written informed consent for the subsequent examination. She complained about the presence of a mass on her palate, which was otherwise asymptomatic, without being able to pinpoint when the tumor initially emerged (Figure 1).

**Figure 2:**
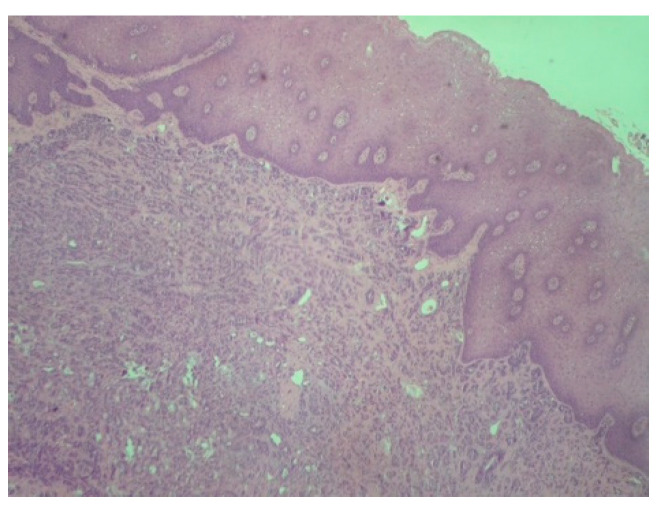
Polymorphous adenocarcinoma: Tumor cells with infiltrative growth pattern in lamina propria. Note the intact overlying squamous epithelium (H-E ×100).

**Figure 3:**
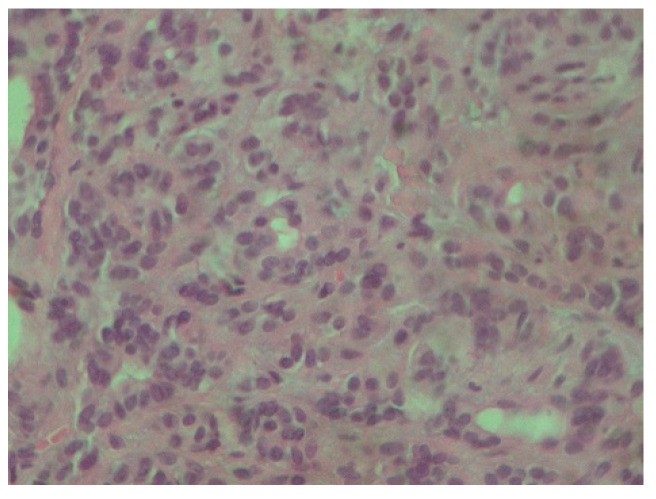
Tumor cells are characterized by small, round nuclei, inconspicuous nucleoli and mild atypia. They grow in adenoid and small trabecular formations (H-E ×400).

**Figure 4:**
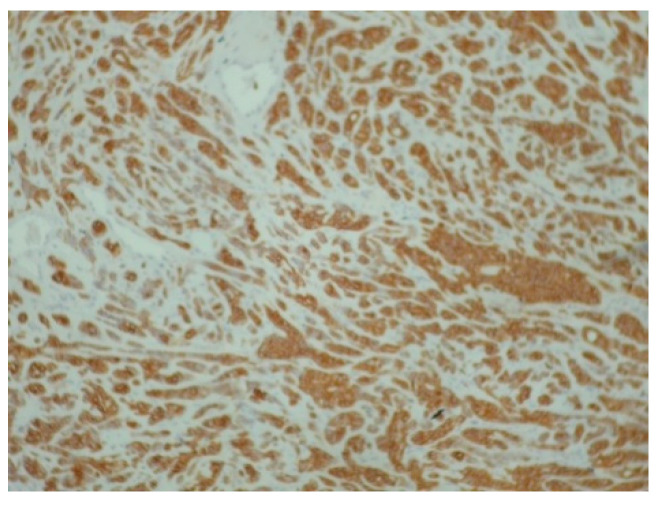
Diffuse and intense positivity of neoplastic cells for cytokeratin 7 (immunoperoxidase ×100).

**Figure 5:**
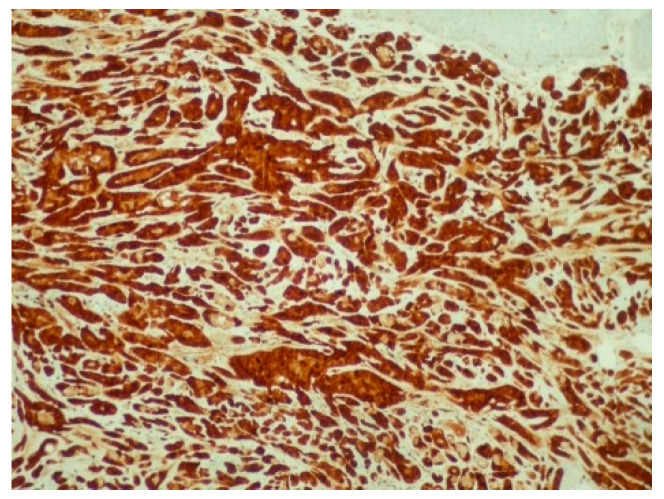
Diffuse and intense positivity of neoplastic cells for S100 protein (immunoperoxidase ×100).

**Figure 6:**
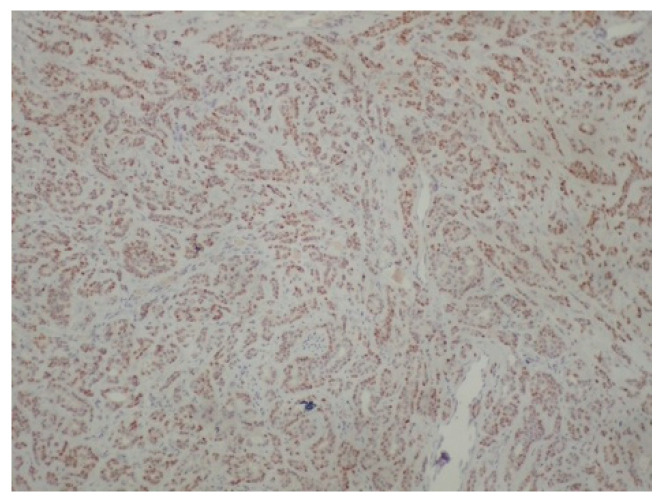
Positivity of neoplastic cells for p63 (immunoperoxidase ×100).

**Figure 7:**
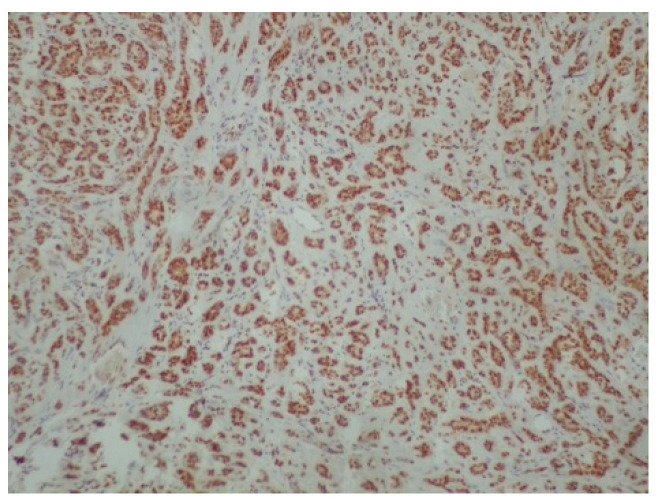
Positivity of neoplastic cells for estrogenic receptors (ER) (immunoperoxidase ×100).

**Figure 8:**
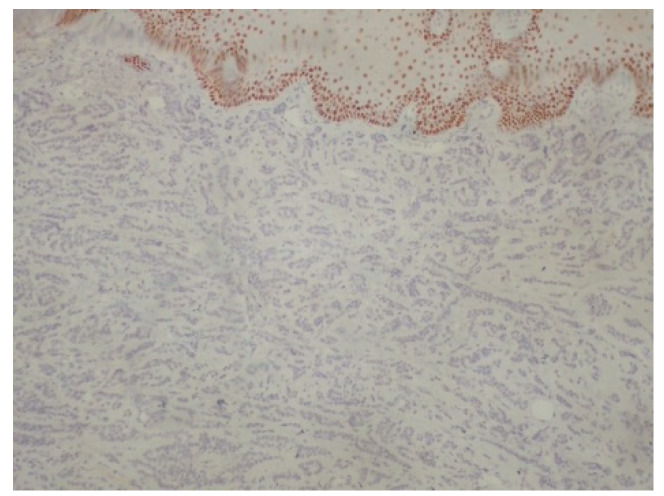
Negativity of neoplastic cells for p40. p63+/p40− immunophenotype is important diagnostic clue for polymorphous adenocarcinoma (immunoperoxidase ×100).

The palpation revealed that the tumor originated from the left half of the soft palate, close to the transition from the soft palate to the hard palate, most probably from the minor salivary glands which are typically localized there. The tumor had a wide base, with a diameter of 2.5 cm, and appeared benign, with regard to its color, texture, and lack of ulceration. Apart from the feeling of having a protuberance in the oral cavity, the patient did not report any other symptoms. However, due to the possible involvement of minor salivary glands and their related pathology, an incisional biopsy was carried out. Biopsy is the golden standard because the alternative, fine needle aspiration, is more likely to lead to misdiagnosis due to insufficient sample volume. The initial differential diagnosis included polymorphous adenocarcinoma, adenoid cystic carcinoma, mucoepidermoid carcinoma and pleiomorphic adenoma. The histopathological examination employed immunohistochemistry to provide a definite diagnosis (Figure 2, Figure 3, Figure 4, Figure 5, Figure 6, Figure 7 and Figure 8).

Following the definitive histopathological diagnosis, the patient was referred to the Department of Oral and Maxillofacial Surgery.

## 3. Discussion

Successfully diagnosing oral polymorphous adenocarcinoma requires specific immunohistochemical markers. One such marker, p63, is frequently positive, and studies have indicated a 100% positivity rate; however, p63 also shows frequent positivity in both adenoid cystic carcinomas and pleomorphic adenomas [3]. p40 immunostaining emerges as a crucial tool, as it is effective in distinguishing polymorphous adenocarcinoma from adenoid cystic carcinomas and pleomorphic adenomas, with all polymorphous adenocarcinoma cases in the study showing negative results for p40 (as in our case) [3]. Furthermore, uniformly positive vimentin and cytokeratin 7 (as in our case) staining can also suffice for a final diagnosis, albeit these markers may not be informative in cases involving rare two-layer ducts [4]. Estrogen receptors are also expected to be negative in pleomorphic adenomas, Warthin’s tumors, mucoepidermoid carcinomas and adenoid cystic carcinomas [5,6]. The expression of hormone receptors in salivary malignancies may vary [7], and one study also shows negative results regarding the presence of estrogen receptors in polymorphous adenocarcinoma [8]. This may be interpreted as follows: estrogen receptors do not belong to the strict immunophenotype of polymorphous adenocarcinoma; however, when positive, screening and imaging of the breast should take place. In our case, the mother of our patient had a medical history of breast cancer, and due to this association between breast cancer and salivary gland cancer [8], the patient was advised to consult a gynecologist. A detailed examination of histological features enables the accurate identification of tumor type, stage, and grade. Diagnosing oral polymorphous adenocarcinoma, especially from adenoid cystic carcinoma, is important, as the management strategies for these tumors differ significantly [9]. Adenoid cystic carcinoma often requires more aggressive treatment approaches like radical neck dissection and adjuvant radiotherapy, which are not typically necessary for oral polymorphous adenocarcinoma [9]. In the case of limited biopsy material, due to the localization in cases such as ours, the oral and maxillofacial pathologists may struggle to obtain sufficient tissue to make a definitive diagnosis [9]. Furthermore, the tissue specimen should be taken from the center of the lesion to depict accurately the histological characteristics of the palatal tumors such as salivary gland neoplasms and lymphomas. Surgical excision with clear margins remains the cornerstone of management of the disease, aiming to reduce the risk of local recurrence, rarely even decades post-diagnosis, or the risk of uncontrolled metastases, which may take place much later than initially expected [10,11]. The guidelines recommend follow-up lasting 10 years or more for effective management of the disease [12]. Radiation therapy may be considered as an adjunct to surgery, particularly if surgical margins are positive or if the tumor localization does not allow for its complete surgical resection [13]. This approach depends also on the tumor’s histopathological complexity, since it may complicate diagnosis, and necessitate aggressive initial treatment [14]. Factors such as tumor size, depth of invasion, and extent of lymph node involvement significantly influence the surgical approach [15]. A recent retrospective study of minor salivary gland tumors reported polymorphous adenocarcinoma as the most common entity (12/30 patients). Most of the patients manifested well-differentiated tumors. A total of 29/30 patients were treated through surgery, 11/30 received adjuvant radiotherapy, and 6/30 received chemotherapy. The relatively high recurrence rate of 26.66% was registered [16]. In our case, the prompt biopsy and definitive surgical treatment elevates the possibility of avoiding any recurrence, and ensuring that the patient will enjoy a disease-free future. This case further illustrates the importance of immunohistochemistry and the need to biopsy even benign-appearing lesions.

## 4. Conclusions

Oral polymorphous adenocarcinoma is a rare clinical entity in the spectrum of salivary gland neoplasms. It typically involves the oral cavity, particularly affecting the minor salivary glands. The clinical presentation is that of a painless, slow-growing mass that may be mistaken for other benign lesions. Accurate diagnosis relies on a combination of histopathological evaluation and imaging. The standard therapeutic approach primarily involves surgical excision. The goal is to achieve optimal patient outcome while minimizing unnecessary morbidity. As surgical techniques and understanding of the disease continue to advance, it is crucial for healthcare providers to stay informed and integrate these developments into practice to improve treatment outcomes for patients.

## Data Availability

The original data presented in the study are included in the article, further inquiries can be directed to the corresponding author.
